# Excess mortality and shortened life expectancy in people with major mental illnesses in Taiwan

**DOI:** 10.1017/S2045796020000694

**Published:** 2020-08-14

**Authors:** Yi-Ju Pan, Ling-Ling Yeh, Hung-Yu Chan, Chin-Kuo Chang

**Affiliations:** 1 Department of Psychiatry, Far Eastern Memorial Hospital, New Taipei City, Taiwan; 2 Institute of Public Health, National Yang-Ming University School of Medicine, Taipei City, Taiwan; 3 Graduate School of Humanities and Social Sciences, Dharma Drum Institute of Liberal Arts, New Taipei City, Taiwan; 4 Department of General Psychiatry, Taoyuan Psychiatric Center, Taoyuan City, Taiwan; 5 Department of Psychiatry, National Taiwan University Hospital and College of Medicine, National Taiwan University, Taipei City, Taiwan; 6 Global Health Program, College of Public Health, National Taiwan University, Taipei City, Taiwan; 7 Institute of Epidemiology and Preventive Medicine, College of Public Health, National Taiwan University, Taipei City, Taiwan; 8 Department of Psychological Medicine, King's College London (Institute of Psychiatry, Psychology and Neuroscience), London, UK

**Keywords:** bipolar disorder, depression, excess mortality, life expectancy at birth, schizophrenia, standardised mortality ratio

## Abstract

**Aims:**

Given the concerns of health inequality associated with mental illnesses, we aimed to reveal the extent of which general mortality and life expectancy at birth in people with schizophrenia, bipolar disorder and depressive disorder varied in the 2005 and 2010 nationally representative cohorts in Taiwan.

**Methods:**

Two nationally representative samples of individuals with schizophrenia, bipolar disorder and depressive disorder were identified from Taiwan's national health insurance database in 2005 and 2010, respectively, and followed-up for consecutive 3 years. The database was linked to nationwide mortality registry to identify causes and date of death. Age-, gender- and cause-specific mortality rates were generated, with the average follow-up period of each age- and gender-band applied as ‘weighting’ for the calculation of expected number of deaths. Age- and gender-standardised mortality ratios (SMRs) were calculated for these 3-year observation periods with Taiwanese general population in 2011/2012 as the standard population. The SMR calculations were then stratified by natural/unnatural causes and major groups of death. Corresponding life expectancies at birth were also calculated by gender, diagnosis of mental disorders and year of cohorts for further elucidation.

**Results:**

The general differential in mortality rates for people with schizophrenia and bipolar disorder remained wide, revealing an SMR of 3.65 (95% confidence interval (CI): 3.55–3.76) for cohort 2005 and 3.27 (3.18-3.36) for cohort 2010 in schizophrenia, and 2.65 (95% CI: 2.55–2.76) for cohort 2005 and 2.39 (2.31-2.48) for cohort 2010 in bipolar disorder, respectively. The SMRs in people with depression were 1.83 (95% CI: 1.81–1.86) for cohort 2005 and 1.59 (1.57-1.61) for cohort 2010. SMRs due to unnatural causes tended to decrease in people with major mental illnesses over the years, but those due to natural causes remained relatively stable. The life expectancies at birth for schizophrenia, bipolar disorder and depression were all significantly lower than the national norms, specifically showing 14.97–15.50 years of life lost for men and 15.15–15.48 years for women in people with schizophrenia.

**Conclusions:**

Compared to general population, the differential in mortality rates for people with major mental illnesses persisted substantial. The differential in mortality for unnatural causes of death seemed decreasing over the years, but that due to natural causes remained relatively steady. Regardless of gender, people with schizophrenia, bipolar disorder and depression were shown to have shortened life expectancies compared to general population.

## Introduction

In comparison with the general population, mortality risk is found much higher for individuals with schizophrenia, bipolar disorder and depression (Osby *et al*., 2001; Hoang *et al*., 2011; Wahlbeck *et al*., 2011; Høye *et al*., 2016; Pratt *et al*., 2016). People with schizophrenia were reported to experience a 2.5-fold risk of death, and a fairly high risk in death of suicide, with around 12-fold relative risk (Saha *et al*., 2007). Standardised mortality ratios (SMRs) for individuals with bipolar disorder were almost double the general population (Chang *et al*., 2010). Research on unipolar depressive disorder showed that the SMRs for all-natural causes of death were around 1.5 and the SMRs for suicide were over 20 (Osby *et al*., 2001). Similarly, research on life expectancy among people with these mental illnesses showed substantially lower longevity, compared to the general population. A UK study revealed 14.6 years of life lost for men and 9.8 years lost for women for patients with schizophrenia (Chang *et al*., 2011). The differential in mortality and shorted life expectancy have been considered as indicators of health inequality that people with mental illnesses did not equally benefit from social and healthcare advancement experienced by the general population.

Our previous study in Taiwan revealed that, from 2003 through 2011, the differential in mortality for people with schizophrenia slightly decreased, whereas the differential in mortality for bipolar disorder individuals remained relatively stable (Pan *et al*., 2017). The discrepant findings on excess mortality, defined as the number of deaths which occurred for a given condition or disease above what we would have expected to see under normal situations, of schizophrenia and bipolar disorder urged further research across various major mental illnesses, including unipolar depression. Despite the need of identifying target population and specific disease or cause to work with, a lack of clarity exists regarding the contributions from specific major causes of death, including cardiovascular disease (CVD), diabetes mellitus, cancer or suicide to the elevated mortality across major mental illnesses. Various causes of death for the issues of health inequalities may reflect discrepancies in access to healthcare system or differences with regards to the burden of healthcare system for people with specific mental disorders. Furthermore, evidence remains relatively scarce for life expectancy at birth for people with these mental illnesses in Taiwan.

Having been defined as ‘severe mental disorders’ (i.e. psychosis – mainly schizophrenia, bipolar disorder and moderate to severe depression) according to the World Health Organization, people with these mental disorders tend to die earlier than the general population with a 10–25 year life expectancy reduction (World Health Organization, 2019). The primary aim of this study was to investigate how the differential of general mortality and life expectancy of people with schizophrenia, bipolar disorder and depressive disorder had changed over the years of observation. The second aim of this study was to explore whether the changes of the excess mortality would differ across diagnosis groups and causes of death to identify potential vulnerable groups. Therefore, we presented the changes of excess mortality and life expectancy at birth in people with schizophrenia, bipolar disorder and depressive disorder, using nationwide cohorts who were diagnosed and treated in 2005 and 2010 in Taiwan, respectively. In our previous analysis (Pan *et al*., 2017), we applied the time frames of observation to define these cohorts for the reason that, earlier than 2005, national health insurance data linking to national mortality registry was lack of basic demographic information and the most updated data available was only given until the end of 2013. For the purposes of consistency, we defined the study cohorts in exact the same way. Three-year SMRs were calculated for each of the defined cohorts using the claim data linked to the national mortality registry from the Health and Welfare Data Science Center, Ministry of Health and Welfare, Taiwan and then stratified by age, sex and diagnosis groups. Beyond general mortality, the SMRs by specific causes were carried out by sex and disease groups of major causes of death. To obtain a more intuitive impression of general mortality for individuals with these mental illnesses, life expectancies at birth were also calculated for the defined cohorts.

## Materials and methods

### Settings

Taiwan is an East Asian country with a population of approximately 23 million. In Taiwan, National Health Insurance, instituted in 1995, is a compulsory social insurance system for healthcare services with a single payer, centralising the disbursement to ensure not only low-cost but also equal access to health services for every citizen and legally hired foreigner who works in Taiwan. In 2008, a total of 22.92 million individuals were involved in the health insurance system, covering 99% of people in this country (National Health Insurance Administration, Taiwan, 2019). The National Health Insurance Research Database (NHIRD) consists of full records of health service utilisation, including demographics, procedures and medication, attached with corresponding health service expenditures. In the database, diagnosis is given by the International Classification of Diseases, 9th revision, clinical modification (ICD-9-CM). For the purposes of research, NHIRD was made available but restrictedly approved by the Health and Welfare Data Science Center, Ministry of Health and Welfare, Taiwan, with a linkage to national mortality registry database to retrieve regularly updated data on causes and date of death.

### Calculation of standardised mortality ratios (SMRs)

To be eligible as subjects of our 2005 and/or 2010 cohorts, the cohort members were ever given a diagnosis of schizophrenia (ICD-9-CM codes: 295*), bipolar disorder (ICD-9-CM codes: 296.0, 296.1, 296.4–296.7) or depression (ICD-9-CM codes: 296.2, 296.3, 300.4, 311) in the year of 2005 or 2010, aged 15 years or over on the index date of diagnosis, and then followed up after their psychiatric diagnosis through the observation periods (i.e. 2005–2008 and 2010–2013). Since the cohorts mixed with newly incident and existing cases for mental disorders, separately by cohorts, we calculated age- and sex-SMRs as the indicator of relative risk of general mortality during the 3-year observational periods for schizophrenia, bipolar disorder and depression, with the 2011/2012 general population of Taiwan as the standard population (Department of Statistics, Ministry of Health and Welfare, Taiwan). First of all, age- and sex-specific mortality rates for these study cohorts were generated by 5-year age bands (i.e. 15–19, 20–24, 25–29, … 85–89 and 90+ years old) and sex. Since the age- and sex-specific mortality rates of general population (i.e. the ‘standard population’ in the calculation process of indirect standardisation) were given by year, we had to apply a ‘weighting’ by the average follow-up period of each age-and-sex band for the calculation of expected number of deaths for the defined cohort (i.e. the ‘target populations’, the group of people with specific mental disorders in our analysis) by summing the weighted number of study subjects in each stratum, timed by their corresponding age- and sex-specific mortality rates of the general population. The observed number of death in a specific study cohort during the observation period of 3 years was then divided by the expected number of deaths to yield an SMR (Roberts *et al*., 2016). Method for the estimation of 95% confidence interval (CI) for an SMR was detailed elsewhere (Higham *et al*., 2005). In addition, we calculated SMRs by three age groups (15–44, 45–64 and 65+ years old), gender, as well as cause of deaths (natural and unnatural), respectively. Unnatural causes of death included ‘accidents and adverse effects’ (ICD-10 codes: E800–E949), ‘homicide and injury purposely inflicted by other persons’ (E960–E969) and ‘suicide and self-inflicted injury’ (E950–E959) in current analysis. We also calculated SMRs for specific underlying causes of death, including cardiovascular disease (CVD), diabetes mellitus, cancer and suicide, respectively.

### Life expectancy at birth

We utilised life expectancy at birth as an alternative but intuitive approach to delineate the extent to which major mental illness impact the general mortality of people with it in Taiwan and the differential in mortality between these people and general population. Life expectancy at birth is a demographic index to describe the general mortality of a specifically defined cohort followed up over a period of time, emphasising the impact of deaths at a younger age. The well accepted approach of its calculation was using the abridged life table method for 5-year age bands to estimate accumulated person-years contributed by the members of specific cohort, then divided by total number of people in the cohort (Chiang, 1984). As what had been done for the calculation of SMRs, ‘weighting’ of the average follow-up period of each 5-year age group in the calculations by gender was adopted to cope with the issue of dynamic study cohorts for an up to 3 years of follow-up period. As a standard template of calculation for life expectancy at birth, an official document of methodological recommendations, published by the UK Office for National Statistics (Methodology Group at Office for National Statistics, 2003), was the basis of related government reports. By using Microsoft Excel, life expectancies at birth for the study cohorts with specific mental disorders were estimated with the mortality rates for Taiwanese people under 15 years old as replacement, since people under 15 years of age were not supposed to be diagnosed as serious mental illnesses or depression in clinical practice. After the age of 15 years old, people with specific mental disorders took over the contribution for their person-time. We compared the outcomes of life expectancy at birth for our study cohorts to the national norms of Taiwan by gender as parameters, at last. All statistical analyses were performed using Stata SE 15 (Chicago, IL, USA) and Excel (Seattle, WA, USA). Alpha level was set at 0.05 as the criteria for statistical significance.

## Results

Table 1 shows that a total of 95 632 people with schizophrenia, 45 392 people with bipolar disorder and 395 006 people with depressive disorders met the inclusion criteria of cohort 2005, and 104 561 people with schizophrenia, 58 317 people with bipolar disorder and 435 585 people with depressive disorders met the inclusion criteria for cohort 2010. The mean age of cohort 2005 was 47.45 years old and that of cohort 2010 was 49.95 years old. Among the calendar cohorts, 39.82–40.89% were men and 3.23–4.58% of them were from low-income households (recognised by the government as total income per person in the household lower than the minimum cost of living, set at 60% of the average monthly per capita nonproductive expenditure during the past year). Individuals with schizophrenia were more likely to be men and from low-income households compared with those with diagnosis of mood disorders.

**Table 1.** Demographic characteristics and physical/mental comorbidities during the 12-month post-index period in 2005/2010 cohorts of major mental illnesses in Taiwan

Variables	Mean ± standard deviation/Number (% in columns)					
	Schizophrenia		Bipolar disorder		Depression	
	Cohort 2005 (*n* = 95 632)	Cohort 2010 (*n* = 104 561)	Cohort 2005 (*n* = 45 392)	Cohort 2010 (*n* = 58 317)	Cohort 2005 (*n* = 395 006)	Cohort 2010 (*n* = 435 585)
Age	42.3 ± 13.4	44.8 ± 13.2	44.8 ± 16.4	46.7 ± 16.0	49.0 ± 17.6	51.6 ± 17.1
Male	51 535 (53.9)	54 953 (52.2)	19 706 (43.4)	24 137 (41.4)	147 916 (37.4)	159 213 (36.6)
Low income household	9342 (9.8)	13 406 (12.8)	1581 (1.4)	2924 (5.0)	6389 (1.6)	11 094 (2.5)
Insurance premium						
⩽17 280 NTD	44 155 (62.8)	74 017 (70.9)	13 086 (42.0)	33 232 (57.3)	91 726 (33.7)	222 461 (51.4)
17 281–36 300 NTD	24 123 (34.3)	27 626 (26.5)	14 922 (47.9)	20 040 (34.5)	143 166 (52.6)	160 858 (37.1)
36 301–72 800 NTD	1915 (2.7)	2595 (2.5)	2831 (9.1)	4325 (7.5)	33 220 (12.2)	43 511 (10.0)
⩾72 801 NTD	97 (0.2)	128 (0.1)	303 (1.0)	445 (0.8)	4202 (1.5)	6228 (1.4)
Owning a catastrophic illness card	71 929 (75.2)	79 685 (76.2)	23 061 (50.8)	29 429 (50.5)	60 452 (15.3)	69 454 (15.9)
Comorbid physical illness						
Cancer	1454 (1.5)	2208 (2.1)	1383 (3.0)	2258 (3.9)	17 926 (4.5)	23 605 (5.4)
CVD	10 353 (10.8)	12 576 (12.0)	9069 (20.0)	11 446 (19.6)	110 885 (28.1)	115 547 (26.5)
COPD	8542 (8.9)	10 178 (9.7)	5925 (13.1)	7335 (12.6)	58 474 (14.8)	56 886 (13.1)
Diabetes mellitus	8761 (9.2)	12 791 (12.2)	5330 (11.7)	8226 (14.1)	48 427 (12.3)	61 890 (14.2)
Hyperlipidaemia	6471 (6.8)	11 292 (10.8)	5000 (11.0)	8871 (15.2)	53 141 (13.5)	78 183 (17.9)
Hypertension	12 341 (12.9)	17 237 (16.5)	9634 (21.2)	13 944 (23.9)	110 246 (27.9)	132 551 (30.4)
Renal disease	2876 (3.0)	3135 (3.0)	2173 (4.8)	2756 (4.7)	22 756 (5.8)	24 356 (5.6)
Comorbid mental illness						
Substance-related disorders	4731 (4.9)	4553 (4.4)	3123 (6.9)	4429 (7.6)	19 319 (4.9)	20 377 (4.7)
Alcohol-related disorder	1080 (1.1)	921 (0.9)	730 (1.6)	936 (1.6)	4009 (1.0)	4464 (1.0)

NTD, new Taiwan dollars. CVD, cardiovascular disease. COPD, chronic obstructive pulmonary disease.

**Fig. 1.**
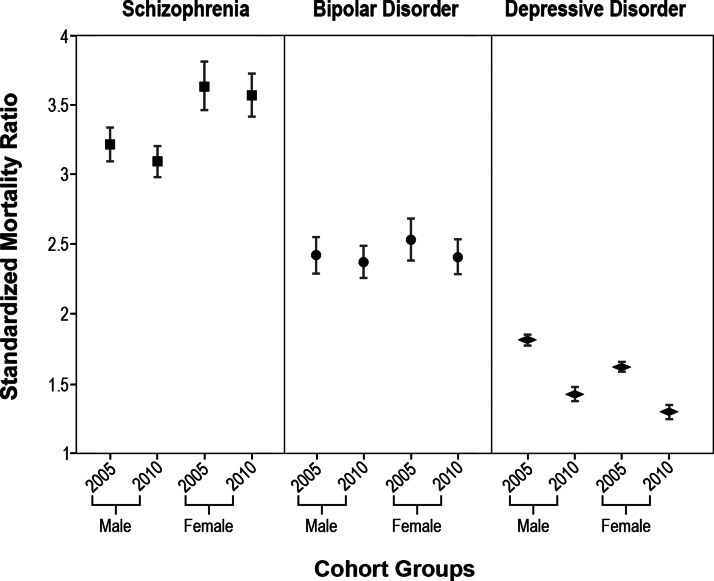
Standardised mortality ratios for people with schizophrenia, bipolar disorder or depression disorder in Taiwan by gender in 2005/2010 cohorts.

The SMRs for people with schizophrenia were over three folds of the general population, showing 3.65 (95% CI: 3.55–3.76) for cohort 2005 and 3.27 (95% CI: 3.18–3.36) for cohort 2010. A visualised summary for SMRs by psychiatric diagnoses, gender, and calendar cohorts was shown in Figure 1. The SMR due to natural causes remained relatively stable as a whole, whereas the SMR due to unnatural causes appeared to drop substantially (2005: 8.25 (95% CI: 7.77–8.75); 2010: 5.79 (95% CI: 5.41–6.18), shown in Table 2). For individuals with bipolar disorder, the SMR for general mortality were 2.65 (95% CI: 2.55–2.76) for cohort 2005 and 2.39 (95% CI: 2.31–2.48) for cohort 2010, respectively. The SMRs due to natural causes in individuals with bipolar disorder remained nearly unchanged over the years whereas the SMRs due to unnatural causes seemed to slightly decrease from 9.40 (95% CI: 8.67–10.16) for cohort 2005 to 7.90 (95% CI: 7.32–8.52) for cohort 2010 (Table 3). In contrast, the SMRs due to natural and unnatural causes decreased simultaneously for those of depressive disorders with main contributions from the lowered mortality of those older than 45 years (Table 4). With regards to SMRs due to specific cause of death, individuals with depression experienced minimising differential in mortality due to cancer, CVD and diabetes mellitus. For those with schizophrenia, the SMRs due to diabetes mellitus slightly reduced over the years. Additionally, the differential in mortality rates due to suicide reduced for schizophrenia and depressive disorder over the years, but not for bipolar disorder, which even showed a potentially worsen scenario for suicide death (Table 5).

**Table 2.** SMRs for people with schizophrenia in cohorts 2005 and 2010 in Taiwan by causes of death, age groups and gender

Causes of death, age groups and gender			Cohort 2005 (*n* = 95 632)					Cohort 2010 (*n* = 104 561)				
			Total number	Number of deaths in 3-year follow-up	SMR (95% CI)			Total number	Number of deaths in 3-year follow-up	SMR (95% CI)		
					Whole group	Age group	Age and gender			Whole group	Age group	Age and gender
All causes	15–44	Male	32 600	1015	3.65 (3.55–3.76)	6.41 (6.09–6.74)	5.40 (5.08–5.75)	28 940	760	3.27 (3.18–3.36)	4.96 (4.67–5.26)	4.26 (3.96–4.57)
		Female	23 453	501			10.3 (9.41–11.24)	22 470	364			7.55 (6.79–8.36)
	45–64	Male	16 126	959		3.53 (3.36–3.7)	3.00 (2.82–3.2)	23 085	1475		3.56 (3.42–3.70)	3.08 (2.92–3.24)
		Female	17 210	699			4.64 (4.3–4.99)	22 799	978			4.64 (4.36–4.94)
	≥65	Male	2809	820		2.56 (2.42–2.69)	2.71 (2.53–2.9)	2928	779		2.34 (2.22–2.46)	2.45 (2.28–2.63)
		Female	3434	556			2.36 (2.16–2.56)	4339	694			2.22 (2.06–2.40)
Natural	15–44	Male	32 600	427	3.30 (3.19–3.42)	4.60 (4.25–4.98)	4.11 (3.73–4.51)	28 940	378	3.23 (3.13–3.33)	4.12 (3.77–4.48)	3.74 (3.37–4.13)
		Female	23 453	182			6.44 (5.54–7.44)	22 470	155			5.46 (4.64–6.40)
	45–64	Male	16 126	728		3.39 (3.2–3.58)	2.91 (2.7–3.13)	23 085	1199		3.58 (3.42–3.74)	3.17 (2.99–3.35)
		Female	17 210	538			4.35 (3.99–4.73)	22 799	776			4.48 (4.17–4.80)
	≥65	Male	2809	782		2.86 (2.7–3.01)	3.01 (2.8–3.23)	2928	761		2.65 (2.51–2.79)	2.79 (2.59–2.99)
		Female	3434	533			2.66 (2.44–2.89)	4339	665			2.51 (2.32–2.71)
Unnatural	15–44	Male	32 600	463	8.25 (7.77–8.75)	10.86 (10.08–11.69)	8.8 (8.02–9.64)	28 940	287	5.79 (5.41–6.18)	7.48 (6.80–8.20)	6.02 (5.34–6.76)
		Female	23 453	249			19.24 (16.93–21.79)	22 470	163			13.03 (11.10–15.19)
	45–64	Male	16 126	191		6.85 (6.13–7.62)	5.51 (4.75–6.35)	23 085	232		5.65 (5.10–6.23)	4.61 (4.04–5.25)
		Female	17 210	141			10.21 (8.59–12.04)	22 799	158			8.40 (7.14–9.82)
	≥65	Male	2809	34		2.87 (2.16–3.73)	2.93 (2.03–4.09)	2928	13		1.63 (1.14–2.25)	1.05 (0.56–1.80)
		Female	3434	21			2.77 (1.72–4.24)	4339	23			2.36 (1.50–3.54)

**Table 3.** SMRs for people with bipolar disorder in cohorts 2005 and 2010 in Taiwan by causes of death, age groups and gender

Causes of death, age groups and gender			Cohort 2005 (*n* = 45 392)					Cohort 2010 (*n* = 58 317)				
			Total number	Number of deaths in 3-year follow-up	SMR (95% CI)			Total number	Number of deaths in 3-year follow-up	SMR (95% CI)		
					Whole group	Age group	Age and gender			Whole group	Age group	Age and gender
All causes	15–44	Male	10 541	368	2.65 (2.55–2.76)	8.00 (7.39–8.64)	6.82 (6.14–7.55)	11 162	371	2.39 (2.31–2.48)	7.09 (6.55–7.65)	6.17 (5.56–6.83)
		Female	13 137	270			10.48 (9.26–11.8)	15 452	278			8.83 (7.82–9.93)
	45–64	Male	6279	409		3.40 (3.15–3.65)	3.05 (2.76–3.36)	9507	589		3.08 (2.90–3.27)	2.8 (2.58–3.04)
		Female	8843	317			3.98 (3.56–4.45)	13 728	455			3.53 (3.21–3.87)
	≥65	Male	2886	579		1.73 (1.63–1.84)	1.69 (1.55–1.83)	3468	711		1.62 (1.54–1.71)	1.64 (1.52–1.76)
		Female	3706	524			1.78 (1.63–1.94)	5000	698			1.61 (1.49–1.73)
Natural	15–44	Male	10 541	168	2.28 (2.18–2.39)	5.83 (5.14–6.6)	5.85 (5–6.8)	11 162	165	2.13 (2.04–2.22)	4.76 (4.17–5.40)	5.07 (4.33–5.91)
		Female	13 137	85			5.8 (4.64–7.18)	15 452	76			4.19 (3.30–5.24)
	45–64	Male	6279	277		2.73 (2.49–2.99)	2.6 (2.31–2.93)	9507	431		2.62 (2.43–2.82)	2.57 (2.34–2.83)
		Female	8843	192			2.94 (2.54–3.38)	13 728	286			2.69 (2.39–3.03)
	≥65	Male	2886	526		1.86 (1.75–1.98)	1.78 (1.63–1.94)	3468	653		1.77 (1.67–1.86)	1.75 (1.62–1.89)
		Female	3706	488			1.95 (1.78–2.14)	5000	655			1.78 (1.65–1.92)
Unnatural	15–44	Male	10 541	164	9.40 (8.67–10.16)	13.52 (12.08–15.1)	10.03 (8.55–11.69)	11 162	159	7.90 (7.32–8.52)	12.19 (10.88–13.60)	9.04 (7.69–10.55)
		Female	13 137	154			21.5 (18.24–25.18)	15 452	159			18.71 (15.91–21.85)
	45–64	Male	6279	116		10.77 (9.42–12.27)	8.41 (6.95–10.09)	9507	136		8.66 (7.68–9.74)	6.44 (5.41–7.62)
		Female	8843	110			15.3 (12.58–18.45)	13 728	146			12.77 (10.78–15.01)
	≥65	Male	2886	47		3.65 (2.89–4.54)	3.58 (2.63–4.76)	3468	55		3.10 (2.48–3.81)	3.42 (2.58–4.45)
		Female	3706	33			3.75 (2.58–5.27)	5000	33			2.68 (1.84–3.76)

**Table 4.** SMRs for people with depressive disorder in cohorts 2005 and 2010 in Taiwan by causes of death, age groups and gender

Causes of death, age groups and gender			Cohort 2005 (*n* = 395 006)					Cohort 2010 (*n* = 435 585)				
			Total number	Number of deaths in 3-year follow-up	SMR (95% CI)			Total number	Number of deaths in 3-year follow-up	SMR (95% CI)		
					Whole group	Age group	Age and gender			Whole group	Age group	Age and gender
All causes	15–44	Male	61 520	1785	1.83 (1.81–1.86)	5.66 (5.46–5.87)	5.38 (5.13–5.63)	56 535	1653	1.59 (1.57–1.61)	5.54 (5.34–5.75)	5.14 (4.89–5.39)
		Female	103 864	1252			6.13 (5.79–6.48)	94 799	1228			6.2 (5.86–6.56)
	45–64	Male	49 669	2851		2.62 (2.55–2.69)	2.61 (2.51–2.71)	63 492	3460		2.35 (2.29–2.41)	2.39 (2.31–2.47)
		Female	90 224	2240			2.64 (2.53–2.75)	115 569	2654			2.3 (2.21–2.38)
	≥65	Male	36 727	7043		1.44 (1.42–1.47)	1.47 (1.44–1.51)	39 186	7543		1.25 (1.23–1.27)	1.31 (1.28–1.34)
		Female	53 002	6191			1.41 (1.38–1.45)	66 004	7422			1.2 (1.17–1.22)
Natural	15–44	Male	61 520	877	1.80 (1.77–1.82)	4.30 (4.07–4.54)	4.86 (4.54–5.19)	56 535	821	1.57 (1.55–1.60)	4.13 (3.90–4.37)	4.6 (4.29–4.92)
		Female	103 864	399			3.44 (3.11–3.79)	94 799	391			3.4 (3.07–3.75)
	45–64	Male	49 669	2170		2.41 (2.33–2.49)	2.49 (2.39–2.6)	63 492	2636		2.15 (2.09–2.21)	2.27 (2.19–2.36)
		Female	90 224	1612			2.3 (2.19–2.42)	115 569	1912			2 (1.91–2.09)
	≥65	Male	36 727	6564		1.58 (1.55–1.61)	1.6 (1.56–1.64)	39 186	7087		1.38 (1.36–1.40)	1.44 (1.40–1.47)
		Female	53 002	5812			1.56 (1.52–1.6)	66 004	6988			1.33 (1.30–1.36)
Unnatural	15–44	Male	61 520	759	5.30 (5.12–5.48)	9.58 (9.1–10.09)	7.84 (7.3–8.42)	56 535	630	4.57 (4.42–4.73)	9.06 (8.58–9.57)	6.98 (6.44–7.54)
		Female	103 864	713			12.55 (11.64–13.5)	94 799	664			12.65 (11.71–13.65)
	45–64	Male	49 669	602		6.20 (5.84–6.57)	5.46 (5.03–5.92)	63 492	710		5.50 (5.21–5.81)	4.97 (4.61–5.35)
		Female	90 224	547			7.28 (6.68–7.91)	115 569	625			6.27 (5.79–6.78)
	≥65	Male	36 727	450		2.59 (2.41–2.78)	2.57 (2.34–2.82)	39 186	422		2.22 (2.07–2.37)	2.14 (1.94–2.35)
		Female	53 002	339			2.62 (2.35–2.92)	66 004	395			2.31 (2.09–2.55)

**Table 5.** Cause-specific SMRs for cohorts 2005 and 2010 in Taiwan by psychiatric diagnoses, major groups of death and gender

Psychiatric diagnosis, major groups of death and gender			Cohort 2005				Cohort 2010			
			Total number	Number of deaths in 3-year follow-up	SMR (95% CI)		Total number	Number of deaths in 3-year follow-up	SMR (95% CI)	
					Whole group	Gender group			Whole group	Gender group
Schizophrenia	Cancer	Male	51 535	276	1.30 (1.19–1.42)	1.12 (0.99–1.26)	54 953	386	1.37 (1.27–1.47)	1.23 (1.11–1.36)
		Female	44 097	241		1.61 (1.41–1.82)	49 608	311		1.58 (1.41–1.77)
	CVD	Male	51 535	385	3.16 (2.92–3.41)	2.96 (2.67–3.27)	54 953	460	3.02 (2.80–3.24)	2.92 (2.66–3.20)
		Female	44 097	244		3.53 (3.10–4.00)	49 608	293		3.18 (2.82–3.56)
	DM	Male	51 535	122	4.18 (3.7–4.71)	3.51 (2.92–4.19)	54 953	132	3.23 (2.86–3.64)	3.03 (2.53–3.59)
		Female	44 097	150		4.95 (4.19–5.81)	49 608	141		3.45 (2.90–4.07)
	Suicide	Male	51 535	459	13.79 (12.81–14.82)	12.42 (11.31–13.61)	54 953	330	9.57 (8.80–10.38)	8.04 (7.19–8.95)
		Female	44 097	275		16.90 (14.96–19.02)	49 608	242		12.91 (11.34–14.65)
Bipolar disorder	Cancer	Male	19 706	217	1.34 (1.21–1.48)	1.35 (1.18–1.54)	24 137	274	1.21 (1.10–1.32)	1.29 (1.14–1.45)
		Female	25 686	155		1.32 (1.12–1.54)	34 180	191		1.11 (0.96–1.28)
	CVD	Male	19 706	183	2.08 (1.87–2.31)	1.94 (1.67–2.24)	24 137	245	1.87 (1.70–2.05)	1.96 (1.72–2.22)
		Female	25 686	164		2.26 (1.93–2.64)	34 180	193		1.76 (1.52–2.03)
	DM	Male	19 706	63	2.70 (2.29–3.15)	2.36 (1.82–3.02)	24 137	76	2.21 (1.90–2.56)	2.17 (1.71–2.71)
		Female	25 686	94		2.98 (2.41–3.64)	34 180	104		2.24 (1.83–2.72)
	Suicide	Male	19 706	216	13.79 (12.81–14.82)	12.42 (11.31–13.61)	24 137	229	15.72 (14.38–17.16)	12.28 (10.74–13.98)
		Female	25 686	225		16.9 (14.96–19.02)	34 180	273		20.54 (18.18–23.13)
Depression	Cancer	Male	147 916	2824	1.52 (1.48–1.56)	1.56 (1.50–1.62)	159 213	3022	1.36 (1.33–1.40)	1.43 (1.38–1.48)
		Female	247 090	2245		1.48 (1.42–1.54)	276 372	2596		1.30 (1.25–1.35)
	CVD	Male	147 916	1850	1.57 (1.52–1.62)	1.61 (1.54–1.68)	159 213	1906	1.27 (1.23–1.31)	1.36 (1.30–1.42)
		Female	247 090	1578		1.53 (1.45–1.6)	276 372	1756		1.19 (1.13–1.25)
	DM	Male	147 916	647	1.96 (1.86–2.06)	2.00 (1.85–2.17)	159 213	583	1.36 (1.29–1.44)	1.53 (1.41–1.66)
		Female	247 090	877		1.93 (1.81–2.06)	276 372	790		1.26 (1.17–1.35)
	Suicide	Male	147 916	1164	10.81 (10.38–11.25)	9.63 (9.08–10.2)	159 213	1128	9.47 (9.10–9.86)	8.34 (7.86–8.84)
		Female	247 090	1218		12.25 (11.57–12.95)	276 372	1255		10.80 (10.21–11.41)

CVD, cardiovascular diseases; DM, diabetes mellitus.

As shown in Table 6, life expectancies at birth for schizophrenia, bipolar disorder and depression were all significantly lower than those of the general population in Taiwan. Specifically, the life expectancies at birth for schizophrenia were 59.53 (57.83-61.22) years for men and 65.65 (63.90-67.39) years for women in cohort 2005; the figures were 60.65 (58.84-62.47) years for men and 67.18 (65.38-68.98) years for women in cohort 2010. Meanwhile, the life expectancies at birth were 74.5 years for men and 80.8 years for women in the general population of Taiwan in 2005. In 2010, the figures were 76.15 years for men and 82.66 years for women (National Health Insurance Administration, Taiwan, 2018). Accordingly, our results revealed 14.97–15.50 years of life lost for men and 15.15–15.48 years lost for women for schizophrenia. For individuals with bipolar disorder, 14.71–15.53 years of life lost for men and 12.69–12.82 years of life lost for women were reported, whereas, for those with depressive disorder, 11.99–12.43 years lost for men and 6.83–7.29 years lost for women were found.

**Table 6.** Life expectancies at birth for people with major mental disorders by gender and psychiatric diagnoses for cohorts 2005 and 2010 in Taiwan with 3-year follow-up

Psychiatric diagnosis	Sex	Cohort 2005		Cohort 2010	
		Life expectancy at birth (95% CI, number of deaths)	Difference from national norm^a^	Life expectancy at birth (95% CI, number of deaths)	Difference from national norm^b^
Schizophrenia	Male	59.53 (57.83–61.22, *n* = 2794)	−14.97	60.65 (58.84–62.47, *n* = 3014)	−15.50
	Female	65.65 (63.90–67.39, *n* = 1756)	−15.15	67.18 (65.38–68.98, *n* = 2036)	−15.48
Bipolar disorder	Male	59.79 (57.87–61.70, *n* = 1356)	−14.71	60.62 (58.96–62.57, *n* = 1671)	−15.53
	Female	68.11 (66.25–69.97, *n* = 1111)	−12.69	69.84 (67.97–71.71, *n* = 1431)	−12.82
Depression	Male	62.51 (60.80–63.22, *n* = 11 679)	−11.99	63.72 (61.97–65.47, *n* = 12 656)	−12.43
	Female	73.97 (72.29–75.64, *n* = 9683)	−6.83	75.37 (73.66–77.08, *n* = 11 304)	−7.29

a Life expectancy at birth of 2005 in Taiwan: male = 74.5 years; female = 80.8 years.

b Life expectancy at birth of 2010 in Taiwan: male = 76.15 years; female = 82.66 years.

## Discussion

Compared to the general population, the risk of death for people with schizophrenia, bipolar disorder and depression in Taiwan had been elevated, with an over one-and-half-fold relative risk in people with depression, over two-fold relative risk in people with bipolar disorder and over three-fold relative risk in people with schizophrenia. Besides, compared to national norms in Taiwan, all three mental illnesses were associated with substantially lower life expectancies at birth, showing 11.99–15.53 years life lost for men and 6.83–15.48 years life lost for women. Beyond the increase of all-cause mortality, we found that observed relative risks of mortality varied by causes of death and types of mental disorders. SMRs due to natural and unnatural causes both seemed to decrease over the study years for those with depressive disorders, mainly contributed by the decreased mortality of those older than 45 years. However, for people with schizophrenia and bipolar disorder, the differential in mortality due to natural causes remained relatively unchanged over the years. Further analyses on specific causes of death showed that the differential in mortality due to suicide decreased over the years for schizophrenia and depressive disorder, but not for bipolar disorder.

Interventions and keeping contacts with specialist mental health services were shown effective at decreasing suicide risks in people with early-stage psychosis (Harris *et al*., 2008; Chen *et al*., 2011). Family involvement at early stages might also reduce the risk of unnatural-cause mortality in a 10-year follow-up of people with schizophrenia and other psychoses (Reininghaus *et al*., 2015). The substantial findings of improvement in general mortality about depressive disorder for both genders shown in Fig. 1 suggested systemic improvements occurring in Taiwan during the study periods which might have driven the reduction in natural death risk as what hypothesised before (Dhar and Barton, 2016). Nonetheless, these reductions were not that obvious for those with bipolar disorder and schizophrenia, highlight the importance of developing related strategies to improve health outcomes for those with bipolar disorder and schizophrenia in terms of what is lacking now and how to improve the current situations. Specifically, the reduced unnatural-cause differential in mortality rates in people with schizophrenia in Taiwan might be partly accounted for by the continuous improvement in the quality of care and accessibility to related healthcare services provided by national health insurance and social welfare systems over the study years. Furthermore, several nationwide suicide prevention programmes have been launched for years in Taiwan. Taiwan National Suicide Surveillance System has been established since 2006, making the first effort to universally register suicide attempts at a national level and giving support to individuals with a structured intervention programme that includes brief counselling, psychoeducation and follow-up contacts. This highly integrated aftercare programme was shown to decrease suicidal behaviours and to delay suicide death (Pan *et al*., 2013). Despite all these suicide-prevention strategies, the persistent suicide-related differential in mortality for bipolar disorder patients in comparison with the general population as revealed in current analysis suggests the need for particular risk assessment and prevention of suicide in those with bipolar disorder.

Health inequality was found differed by type of mental illnesses with schizophrenia as the most disadvantaged group showing a remarkably high SMR in the current study. Among the mortality due to specific natural causes, over three-fold relative risk for CVD and diabetes mellitus related mortality warrants further attention in patients with schizophrenia. In contrast to bipolar disorder, over the study years, the SMRs of cancer, CVD and diabetes mellitus all decreased in individuals with depression, suggesting a reduction in the natural-cause differential in mortality rates. However, for those with schizophrenia and bipolar disorder, only the SMR of diabetes mellitus seemed decreasing for patients with schizophrenia and no specific natural death causes apparently reduced for those with bipolar disorder, suggesting the existence of a persistent differential in mortality rates due to comorbid physical conditions. With regards to each specific cause of death, a systematic review showed that the pooled SMR due to cancer was 1.40 (95% CI: 1.29–1.52, *p* < 0.001) for individuals with schizophrenia (Zhuo *et al*., 2017); our results showed an SMR of 1.22 in cohort 2005 and 1.37 in cohort 2010 for cancer related mortality. Prior research also showed that the impact of diabetes mellitus on mortality was significantly higher in schizophrenia than hospital controls (Schoepf *et al*., 2012). A Taiwanese study reported that the adjusted hazard ratios were 1.49 (95% CI: 1.32–1.68) for macrovascular complications and 3.68 (95% CI: 3.21–4.22) for all-cause mortality in people with diabetes and schizophrenia, compared with those with diabetes only (Wu *et al*., 2015). For people with mood disorders, a hospital-based Swedish study reported that, most excess deaths for people with bipolar disorder were from natural causes in comparison with general population, but, for unipolar depressive disorder, most excess deaths were from unnatural causes (Osby *et al*., 2001). Along with our finding of the persistent differential in mortality owing to natural causes, future efforts on prevention, monitoring and management of physical comorbidities is of utmost relevance to improve overall health for vulnerable people with mental illnesses, particularly for those with schizophrenia and bipolar disorder.

Our results revealed that all three major mental illnesses were associated with substantially lower life expectancies at birth, showing 11.99–15.53 years life lost for men and 6.83–15.48 years life lost for women (National Health Insurance Administration, Taiwan, 2018). In comparison with a previous UK study (Chang *et al*., 2011), our results seemed to present more years of life lost particularly for women with schizophrenia and men with bipolar disorder. Prior research revealed that individuals with serious mental illnesses in eight US states from 1997 to 2000 had lower life expectancies of 13 to more than 30 years life lost, compared to the general population (Colton and Manderscheid, 2006). A Swedish study using a nationwide hospital discharge registry reported substantial differences in life expectancy at age 30 in comparison with the general population, including affective psychosis (15.9 years life lost), substance abuse (15.6 years life lost) and organic psychosis (14.8 years life lost) among men and organic psychosis (22.6 years life lost), mental retardation (14.7 years life lost) and substance abuse (18.8 years life lost) among women (Hannerz *et al*., 2001). Preliminary strategies for preventing premature deaths had been suggested, emphasising the management of suicide risks and physical illnesses by the improvement of accessibility to health care for comorbid physical illnesses (Auquier *et al*., 2006). Last but perhaps foremost, intervention programmes regarding the modifications of lifestyle risk factors, including health psychoeducation, regular health screening, intensive management of metabolic syndrome and health promotion programmes, such as encouraging exercise, should be further re-enforced.

### Strengths and limitations

The strengths of current analysis contained great representativeness of whole country coverage, all diagnoses of schizophrenia, bipolar disorder and depressive disorder given in clinical settings, and follow-up for up to consecutive 3 years, generating evidence of great interest to clinicians, investigators and policy makers. Linkage to national mortality registry was also a strength of current study, providing complete all causes of death with sufficient details and exact date of death. Of note, the major limitation was that these two defined cohorts were overlapped to a certain extent (i.e. people with major mental illness may keep alive through these two observation periods), making it challenging to discriminate the differences between two partially dependent cohorts by proper statistical tests. The other one was that the estimations of SMR and life expectancy at birth were just preliminary attempts to control confounding from age and/or gender structure, not a complete solution for all potential confounders. Thus, further analysis with full consideration of all potential confounders by studies in various settings to address the issue of residual confounding is warranted. Finally, because of the issue of limited observation windows, we could only identify the date of the first diagnosis given in 2005/2010, for which cohort members were inevitably mixing incident and prevalent cases. For incident cases, the adjustment period around their confirmed diagnoses could be thus ignored and disregard some at-risk periods.

## Conclusion

In conclusion, based on large nationwide cohorts, our investigation suggested that, compared to the general population, the excess mortality and shortened life expectancy in people with schizophrenia, bipolar disorder and depression remained noticeable in Taiwan. Specifically, we revealed differential patterns for the changes of different mental illnesses. Future studies are warranted to further elucidate relative risks in different causes of deaths and to generate evidence assisting us in reducing mortality risks by improving healthcare services and physical health for those with major mental illnesses. Besides, comorbid psychiatric illnesses might be of specific research interest in future for the finding that suicide prevention strategies seemed less effectively for bipolar disorder than depression or schizophrenia. Further understanding on the differences in theses under-explored mental illnesses with regards to suicide risk may answer the heterogeneity in the nature and intensity of disease dimensions, including the role of impulsivity from a symptomatology perspective.

## Data Availability

For data used in the current study, there are ethical or legal restrictions on sharing de-identified dataset, regulated by the Ministry of Health and Welfare, Taiwan.
